# Multiplex ligation dependent probe amplification (MLPA) for rapid distinction between unique sequence positive and negative marker chromosomes in prenatal diagnosis

**DOI:** 10.1186/1755-8166-4-2

**Published:** 2011-01-14

**Authors:** Diane Van Opstal, Marjan Boter, Petra Noomen, Malgorzata Srebniak, Guus Hamers, Robert-Jan H Galjaard

**Affiliations:** 1 Department of Clinical Genetics, Erasmus Medical Centre, PO Box 2040, 3000 CA Rotterdam, The Netherlands

## Abstract

**Background:**

Small supernumerary marker chromosomes (sSMC) are extra structurally abnormal chromosomes that cannot be unambiguously identified with conventional chromosome banding techniques. These marker chromosomes may cause an abnormal phenotype or be harmless depending on different factors such as genetic content, chromosomal origin and level of mosaicism. When a sSMC is found during prenatal diagnosis, the main question is whether the sSMC contains euchromatin since in most cases this will lead to phenotypic abnormalities. We present the use of Multiplex Ligation Dependent probe Amplification (MLPA) for rapid distinction between non-euchromatic and euchromatic sSMC.

**Results:**

29 well-defined sSMC found during prenatal diagnosis were retrospectively investigated with MLPA with the SALSA MLPA centromere kits P181 and P182 as well as with the SALSA MLPA telomere kits P036B and P070 (MRC Holland BV, Amsterdam, The Netherlands). All unique-sequence positive sSMC were correctly identified with MLPA, whereas the unique-sequence negative sSMC had normal MLPA results.

**Conclusions:**

Although different techniques exist for identification of sSMC, we show that MLPA is a valuable adjunctive tool for rapidly distinguishing between unique-sequence positive and negative sSMC. In case of positive MLPA results, genetic microarray analysis or, if not available, targeted FISH can be applied for further identification and determination of the exact breakpoints, which is important for prediction of the fetal phenotype. In case of a negative MLPA result, which means that the sSMC most probably does not contain genes, the parents can already be reassured and parental karyotyping can be initiated to assess the heritability. In the mean time, FISH techniques are needed for determination of the chromosomal origin.

## Background

The finding of a sSMC presents a challenge in prenatal diagnosis particularly for prediction of the clinical consequences which will depend on its genetic content, familial occurrence, level of mosaicism and chromosomal origin [1-5] and parental origin of the sSMC related sister chromosomes [6]. According to the review of Liehr and Weise [7] sSMC are to be expected in 0.075% of all analysed prenatal cases. In case of fetal ultrasound abnormalities this frequency rises to 0.204%, which is 2.7x higher than in the general prenatal population.

Before the introduction of FISH for cytogenetics, identification studies involved the use of classical staining techniques such as GTG, QFQ, Ag-NOR, CBG and DA-DAPI [1]. Nowadays, different molecular cytogenetic techniques have been developed for identification of sSMC, such as FISH techniques like cenM- and subcenM-FISH [8,9], multicolour banding (MCB)[10], microdissection followed by reverse FISH [11,12], spectral karyotyping (SKY) [13], M-FISH [14] and genomic microarray analysis [15,16]. These techniques are expensive and not available in all cytogenetic laboratories [17].

In this paper we present the use of Multiplex Ligation Dependent Probe Amplification (MLPA) (MRC Holland, Amsterdam, The Netherlands) as an alternative approach for identification of euchromatic sSMC. On the basis of 29 well characterised sSMC we show that MLPA can rapidly distinguish between unique sequence positive and negative sSMC, which is the most important task when finding a sSMC prenatally. However, other molecular cytogenetic techniques will remain necessary for determining the exact genetic content in case of a positive sSMC, whereas FISH techniques will still be indispensible for identification studies in case of an unique sequence negative sSMC.

## Methods

### Samples

We retrospectively tested the value of MLPA for sSMC identification on 29 well-defined sSMC found during prenatal diagnosis in amniotic fluid (AF)(n = 26) and chorionic villi (CV)(n = 3) (see table 1 and additional file 1). For routine cytogenetics GTG-banding was used in all cases according to standard techniques. Mostly, sSMC identification was done with FISH, sometimes after additional staining with DA-DAPI [18] (see additional file 1). In 23/29 cases the sSMC was present in all investigated cells. In 6/29 cases mosaicism was found in cultured CV or AF cells with the level of mosaicism varying between 30 and 89% (table 1).

**Table 1:** 29 well-defined sSMC in AF or CV cell cultures that were used in this study

Case	sSMC	Euchromatin (based on GTG/FISH)^1^	% of cultured cells with sSMC
1	der(3)(:p12.2->cen:)^2^	+	100
2	min(4)(:p11->q11:)	-	100
3	psu idic(9)(q12)	+	87,5
4	i(12)(p10)	+	89
5	i(12)(p10)	+	100
6	neo(12) (pter->p12.3:)	+	47
7	der(13)t(4;13)(q31.3;q13)	+	100
8	min(13 or 21)	-	100
9	min(13 or 21)	-	100
10	inv dup(14)(q11.2)	-	100
11	der(14)t(14;16)(q12;q21)	+	100
12	inv dup(15)(q12)	+	100
13	neo(15)(qtel->q2?4:)^3^	+	100
14	der(15)t(9;15)(p12;q14)	+	100
15	inv dup(15)(q11)	-	100
16	inv dup(15)(q11.2)	-	100
17	inv dup(15)(q11.2)	-	100
18	min(16)(:p11.1->q11.1:)	-	30
19	min(17)(:p11.1->q11.1:)	-	45
20	r(20)(q11.21q13.12)	+	87
21	inv dup(22)(q11.21)	+	100
22	inv dup(22)(q11.21)	+	100
23	inv dup(22)(q11.21)	+	100
24	inv dup(22)(q11.21)	+	100
25	del(22)(q11.2)	+	100
26	inv dup(22)(q11.1)	-	100
27	inv dup(22)(q11.1)	-	100
28	inv dup(22)(q11.1)	-	100
29	inv dup(22)(q11.1)	-	100

^1 ^For identification details, see additional file 1[38]. ^2 ^This case was previously published by Srebniak et al. [39]. ^3^This case was published earlier by Van Opstal et al. [40].

### FISH

Metaphase FISH was performed according to standard techniques. The probes that were used for identification were whole chromosome paints (wcp's)(Kreatech Diagnostics, Ankeveen, The Netherlands and Euro-Diagnostica AB, Nijmegen, The Netherlands), centromere probes (Abbott Molecular Inc., Des Plaines, USA; Resources for Molecular Cytogenetics, Bari, Italy (http://www.biologia.uniba.it/rmc/) and partially received from several investigators), subtelomere-probes [19], locus-specific probes (SNRPN from Cytocell Ltd, Cambridge, UK; LSI TEL AML1 from Abbott Molecular Inc., Des Plaines, USA and others like 102D10 (CES-probe), Y41 and Y11H11 (15q11), r521 (rDNA-probe) were kindly provided by several investigators) and subcentromere-BAC clones that were selected from the University of California Santa Cruz (UCSC) genome browser (http://genome.ucsc.edu) (see additional file 1).

FISH slides were analyzed using the Axioplan 2 Imaging microscope (Zeiss), and images were collected using Isis Software System (Metasystems).

### Sample preparation for MLPA and SNP array

DNA was isolated from 4 ml of uncultured AF or from cultured CV or AF cells. AF cells were cultured by the in situ method and CV were cultured using trypsin-EDTA and collagenase treatment as described previously [20]. DNA-isolation from uncultured AF cells was done with the Chemagic Magnetic Separation Module I (Chemagen, Baesweiler, Germany). DNA isolation from cultured cells was performed using the QIAamp DNA Mini Kit from Qiagen (Hilden, Germany) or Puregene DNA Purification Kit (Qiagen, Hilden, Germany) according to the manufacturer's instructions.

### MLPA-reaction and data analysis

4 SALSA MLPA kits were used: two centromere kits, P181 and P182, and two telomere-kits, P036B and P070 (http://www.mlpa.com/WebForms/WebFormMain.aspx). Between 20 and 70 ng of DNA was used in a MLPA reaction which was performed on a PCR thermocycler with heated lid (Biometra Thermal Cycler, Westburg, The Netherlands). MLPA reaction and data analysis were performed as described earlier [21]. In order to enable the detection of chromosomal mosaicism as was seen in 6/29 cases, we calculated own cut off values (median ± 2x SD) for the different probes on the different chromosomes for all four MLPA-kits on the basis of 95 (P181), 91 (P182), 104 (P036B) and 105 (P070) normal samples (see table 2).

**Table 2:** Cut off values (median probe signal ±2 SD) for the different probes in the MLPA kits P181, P182, P036B and P070

Probes P181	Cut off values (N = 95)		Probes P182	Cut off values (N = 91)	
	Minimum	Maximum		Minimum	Maximum
3p11.2 EPHA3	0,923	1,077	3p11.2 EPHA3	0,923	1,077
3q11.2 PROS1	0,907	1,093	3q11.2 PROS1	0,913	1,087
4p11 OCIAD1	0,880	1,114	4p11 OCIAD1	0,880	1,114
4q12 SGCB	0,946	1,054	4q12 USP46	0,919	1,081
9p13.2 IGFBPL1	0,902	1,098	9p11 EXOSC3	0,940	1,060
9q13 TJP2	0,933	1,067	9q13 TJP2	0,909	1,079
12p11.21 PKP2	0,888	1,112	12p11.21 PKP2	0,894	1,090
12q12 KIF21A	0,899	1,101	12q12 KIF21A	0,886	1,114
13q11 HSMPP8	0,881	1,119	13q11 HSMPP8	0,924	1,060
13q11 ZNF198	0,930	1,066	13q11 ZNF198	0,881	1,119
14q11.2 ADPRTL2	0,863	1,115	14q11.2 ADPRTL2	0,913	1,079
14q11.2 APEX	0,917	1,083	14q11.2 APEX	0,932	1,066
15q11.2 NIPA2	0,883	1,097	15q11.2 NIPA2	0,864	1,128
15q11.2 NDN	0,916	1,078	15q11.2 MKRN3	0,874	1,126
16p11.2 TGFB1I1	0,882	1,098	16p11.2 ERAF	0,927	1,071
16q12 ORC6L	0,914	1,086	16q12 VPS35	0,899	1,101
17p11.2 MAP2K3	0,934	1,064	17p11.2 MAP2K3	0,828	1,170
17q11.1 WSB1	0,927	1,073	17q11.1 WSB1	0,931	1,069
20p11.2 PYGB	0,894	1,102	20p11.21 ZNF337	0,914	1,086
20q11.21 DUSP15	0,912	1,088	20q11.21 REM1	0,893	1,075
21q11 STCH	0,911	1,089	21q11 STCH	0,896	1,104
21q11 SAMSN1	0,861	1,153	21q11 SAMSN1	0,889	1,111
22q11.2 CECR5	0,872	1,086	22q11.2 CECR1	0,877	1,083
22q11.2 CECR1	0,901	1,089	22q11.2 SLC25A18	0,916	1,084

**Probes P036B**	**Cut off values (N = 104)**		**Probes P070**	**Cut off values (N = 105)**	
	**Minimum**	**Maximum**		**Minimum**	**Maximum**

3p CHL1	0.890	1.110	3p CHL1	0.916	1.080
3q BDH	0.905	1.092	3q KIAA0226	0.933	1.067
4p FLJ20265	0.900	1.101	4p ZNF141	0.828	1.172
4q FRG1	0.744	1.239	4q FRG1	0.865	1.143
9p DMRT1	0.897	1.103	9p FLJ00026	0.915	1.083
9q MRPL41	0.847	1.148	9q EU-HMTase1	0.935	1.065
12p SLC6A12	0.947	1.053	12p RBBP2	0.937	1.063
12q ZNF10	0.858	1.126	12q ZNF10	0.909	1.091
13p PSPC1	0.914	1.074	13p PSPC1	0.900	1.116
13q F7	0.883	1.116	13q CDC16	0.923	1.073
14p HEI10	0.919	1.081	14p ADPRTL2	0.929	1.071
14q MTA1	0.859	1.143	14q MTA1	0.906	1.094
15p CYFIP1	0.925	1.075	15p NDN	0.908	1.092
15q ALDH1A3	0.890	1.110	15q FLJ22604	0.938	1.062
16p POLR3K	0.894	1.108	16p DECR2	0.828	1.178
16q GAS11/GAS8	0.894	1.106	16q GAS11	0.936	1.060
17p RPH3AL	0.901	1.099	17p RPH3AL	0.843	1.163
17q TBCD	0.888	1.108	17q SECTM1	0.890	1.096
20p SOX12	0.843	1.157	20p FLJ22115	0.888	1.112
20q OPRL1	0.866	1.134	20q FLJ20517	0.883	1.117
21p RBM11	0.879	1.121	21p STCH	0.894	1.132
21q HMT1	0.853	1.147	21q S100B	0.921	1.065
22p BID	0.894	1.094	22p IL17R	0.837	1.163
22q RABL2B	0.875	1.127	22q ARSA	0.904	1.096

Only the chromosomes from which the sSMC in this paper are derived, are indicated.

### SNP array, data analysis and interpretation

In two cases (cases 13 and 25) a SNP array (HumanCytoSNP-12, Illumina) was performed because of discrepancies between the results of GTG/FISH and MLPA. 200 ng of DNA isolated from cultured cells was used in both cases. DNA amplification, tagging and hybridisation were performed according to the manufacturer's protocol. Array slides were scanned on the iScan Reader (Illumina). Data analysis was performed using Genome Studio version 2010.1 (Illumina). The HapMap control set provided by the manufacturer was used as a control.

## Results

### Unique sequence positive sSMC

All unique sequence positive sSMC (Table 1) were correctly identified with MLPA with the centromere kits (cases 1 and 20), telomere kits (cases 6 and 13) or both (cases 3-5, 7, 11, 12, 14, 21-25) (see table 3) confirming GTG/FISH results. There were no false negative cases.

**Table 3:** MLPA results of 16 prenatal cases with a unique sequence positive sSMC

No	sSMC	DNA source for MLPA	MLPA-results: copy number of sequence(s) in the kit, genes that they target and their abnormal relative probe signal(s)							
			**P181**		**P182**		**P036B**		**P070**	

			**Copy numb.**	**Relative probe signal(s)**	**Copy numb.**	**Relative probe signal(s)**	**Copy numb.**	**Relative probe signal(s)**	**Copy numb.**	**Relative probe signal(s)**

	**Mosaic cases**^1^									
										
3	psu idic(9)(q12) (87,5%)	LTC-CV	4	IGFBPL1: 2.046	4	EXOSC3: 1.9013	4	DMRT1: 1.854	4	FLJ00026: 1.835
4	i(12)(p10) (89%)	uAF	4	PKPR2: 1.577^2^	4	PKP2: 1.520^2^	4	SLC6A12: 1.718	4	RBBP2: 1.602
6	neo(12) (pter->p12.3:) (47%)	uAF	2	PKPR2: 1.006 KIF21A: 1.015	2	PKP2: 1.016 KIF21A: 1.045	4	SLC6A12: 1.765	4	RBBP2: 1.519^2^
20	r(20)(q11.21q13.12) (87%)	uAF	3	DUSP15: 1.461	3	REM1: 1.183^3^	2	SOX12:1.088 OPRL: 1.000	2	FLJ22115: 0.989 FLJ20517: 1.004
	**Non-mosaic cases**									
1	der(3) (:p12.2->cen:)	LTC-CV	3	EPHA3: 1.178^3^	3	EPHA3: 1.226^3^	2	CHL1: 0.974	2	CHL1: 0.965
5	i(12)(p10)	uAF	4	PKPR2: 1.632	4	PKP2: 1.795	4	SLC6A12: 1.645	4	RBBP2: 1.699
7	der(13)t(4;13) (q31.3;q13)	cAF	3	HSMPP8: 1.470 ZNF198: 1.322	3	HSMPP8:1.387 ZNF198: 1.534	3	PSPC1: 1.352 FRG1: 1.235^4^	3	PSPC1:1.495 FRG1: 1.300
11	der(14)t(14;16) (q12;q21)	cAF	3	APEX: 1.358 ADPRTL2:1.310	3	ADPRTL2: 1.371 APEX: 1.410	3	HEI10: 1.393 GAS11/GAS8: 1.498	3	ADPRTL2: 1.462 GAS11: 1.381
12	inv dup(15) (q12)	cAF	4	NDN: 1.702 NIPA2: 1.770	4	MKRN3: 1.635 NIPA2: 1.851	4	CYFIP1: 1.714	4	NDN: 1.668
13	neo(15) (qtel->q2?4:)	cAF	2	NDN: 1.007 NIPA2: 0.932	2	MKRN3: 0.879 NIPA2: 1.087	3	ALDH1A3: 1.450	3	FLJ22604: 1.485
14	der(15)t(9;15) (p12;q14)	uAF	3	NDN: 1.324 NIPA2: 1.406 IGFBPL1: 1.401	3	MKRN3: 1.493 NIPA2: 1.335 EXOSC3: 1.314	3	CYFIP1: 1.424 DMRT1: 1.455	3	NDN: 1.324 FLJ00026: 1.820^5^
21	inv dup(22) (q11.21)	cAF	4	CECR1: 1.831 CECR5: 1.572^6^	4	CECR1: 1.830 SLC25A18: 1.773	4	BID: 1.825	3^6^	IL17R: 1.548
22	inv dup(22) (q11.21)	uAF	4	CECR1: 1.789 CECR5: 1.654	4	CECR1: 1.796 SLC25A18: 1.811	4	BID: 1.907	4	IL17R: 1.881
23	inv dup(22) (q11.21)	uAF	4	CECR1: 1.694 CECR5: 1.724	4	CECR1: 1.711 SLC25A18: 1.691	4	BID: 1.940	4	IL17R: 2.080
24	inv dup(22) (q11.21)	cAF	4	CECR1: 1.840 CECR5: 1.819	4	CECR1: 1.790 SLC25A18: 2.098	4	BID: 1.693	3^6^	IL17R: 1.527
25	del(22) (q11.2)	LTC-CV	4	CECR1: 1.770 CECR5: 1.621	4	CECR1: 1.760 SLC25A18: 1.869	4	BID: 1.688	4	IL17R: 1.806

LTC-CV = long-term cultured chorionic villi; uAF = uncultured amniotic fluid cells; cAF = cultured amniotic fluids cells.

^1^: Level of mosaicism was determined in cultured cells, whereas DNA from uncultured cells was used for MLPA in cases 4, 6 and 20.

^2^: Although relative probe signals indicate 3 copies (<1.6) we interpreted these results as 4 copies because of mosaicism in the cell cultures.

^3 ^relative probe signal is clearly above the normal cut off value but < 1.3, probably indicating mosaicism.

^4 ^the relative probe signal of 4qtel probe (FRG1) is in fact below the cut off value of 1.239. The FRG1 specific probes in the P036 kits were found not to be reliable by the manufacturer due to the presence of population specific SNP's in FRG1 (see website of MRC-Holland).

^5 ^The probe for 9p in the P070 kit was found to be duplicated in some healthy individuals (see MRC Holland-website) which may explain the relative probe signal of 1.820 indicating 4 instead of 3 copies of 9ptel.

^6 ^The relative probe signal of the sequence targeting CECR5 in case 21 and IL17R in cases 21 and 24 is < 1.6 and therefore indicating 3 instead of 4 copies. However, on the basis of the relative probe signals of the other probes in the same and/or other kits, we assume the presence of 4 copies of CECR5 and/or IL17R on the sSMC in cases 21 and 24.

### a. Non-mosaic cases

The relative probe signals in most non-mosaic cases (see table 1) correctly discriminated between 3 and 4 copies of the investigated probes, with relative probe signals > 1.3 and < 1.5 for a trisomy and > 1.6 for a tetrasomy (see table 3). In 3/23 non-mosaic cases a discrepancy was found.

**Case 1**: Although amplification of EPHA3 confirmed the results of FISH and DNA marker studies (sSMC=der(3)(:p12.2->cen:) in case 1, the relative probe signals of the 3p11.2 marker *EPHA3 *in both centromere-kits were not above 1.3 (1.178 in P181 and 1.226 in P182) as would be expected in a full blown case. However, they were clearly above the normal cut off value of 1.077 for both kits probably indicating loss of the sSMC in part of the cells and therefore mosaicism at the time that DNA for MLPA was isolated from the cell cultures.

**Figure 1:**
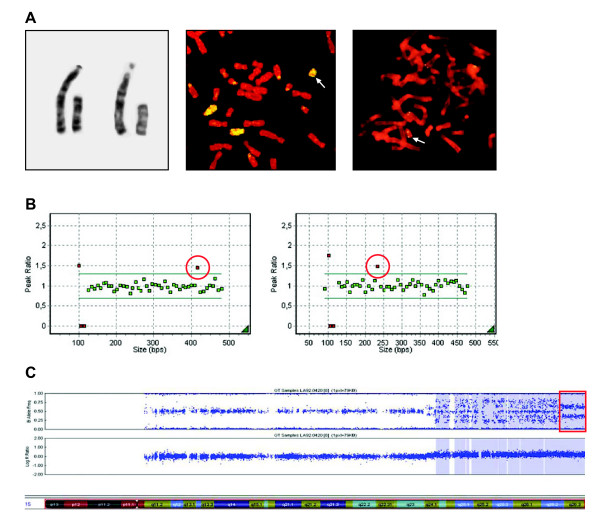
**Identification of the sSMC in case 13 with GTG and FISH (a), MLPA (b) and SNP array analysis (c)**. **a**. GTG and FISH results: left: partial karyotype of 2 cells showing a normal chromosome 15 on the left and the sSMC on the right. Middle: partial metaphase after FISH with WCP15. Three chromosomes, both normal chromosomes 15 and the sSMC (arrow), are stained, proving the chromosome 15 origin of the sSMC. Right: Partial metaphase after FISH with probe p80, located at 15q25-qter [37], showing 2 signals on both ends of the sSMC (arrow) indicative for a neo(15). ***b***. MLPA results with the kits P036B (left) and P070 (right). The probes targeting *ALDH1A3 *and *FLJ22604 *(within red circle), respectively, both located at 15qtel, are clearly elevated but below 1.6 indicating 3 copies of both genes instead of four on the sSMC. **c**. HumanCytoSNP-12 result. Only chromosome 15 is depicted. The upper part shows the B-allele frequency (BAF) and the lower part shows the Log2 intensity along chromosome 15. Based on BAF (absence of a BAF of 0.5 at 15q26.3 while a meiotic origin is likely due to the presence of a BAF of 0.5 in a large part of the sSMC, indicating the presence of a third haplotype) and Log2 ratio (indicating 3 copies of the most distal end of 15q), the MLPA result of a trisomy at the distal q-arm at 15q26.3, is confirmed.

**Case 13 **showed a full blown neo(15) in cultured AF cells (8 cell clones investigated) (see Figure 1a), which is an analphoid sSMC with a neocentromere and consisting of two copies of the distal end of chromosome 15q. However, the relative probe signals of the 15q-subtelomere probes were only 1.45 and 1.428 in respectively P036B and P070, indicative of a trisomy but not a tetrasomy as expected (Figure 1b).

In order to elucidate this discrepancy, genomic microarray analysis was performed. Investigations with the HumanCytoSNP-12 indicate that this case is more complex than initially thought which may explain the MLPA results. Based on Log R ratio and B-allele frequency (BAF), we expect mosaicism of different abnormal cell lines containing different sSMC. However, BAF's of 0, 1, 0.333 and 0.667 and absence of a BAF of 0.5 at 15q26.3 (in contrast to the region 15q24.1-q26.2) indicate a trisomy at 15qtel which confirms the MLPA results (see Figure 1c).

**Figure 2:**
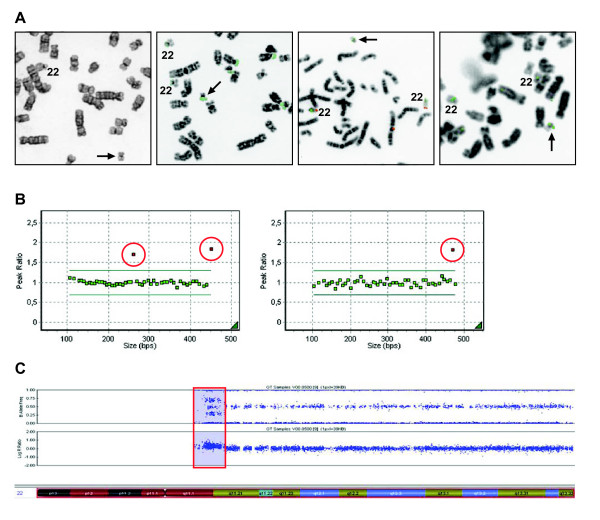
**Identification of the familial sSMC in case 25: GTG and FISH (a), MLPA (b) and HumanCytoSNP-12 (c) results**. **a. **Left. GTG partial metaphase showing the sSMC (arrow). 2^nd ^picture: partial metaphase after FISH with the ribosomal DNA probe r521, showing one signal on the sSMC (arrow) and on the short arm of all acrocentric chromosomes. 3^rd ^picture: partial metaphase after FISH with p190.22 (22cen) (green) and 22qtel probe (red) showing only one centromere signal and no 22qtel signal on the sSMC (arrow). Both normal chromosomes 22 are positive with 22cen as well as with 22qtel probe. Right: partial metaphase after FISH with a Cat Eye Syndrome critical region probe 102D10 showing one signal on the sSMC (arrow) and on both normal chromosomes 22. **b. **MLPA results with centromere kit P182 (left) en telomere kit P070 (right). The relative probe signals of the sequences targeting *CECR1 *and *SLC25A18 *in P182 and *IL17R *in P070 (in red circle) are clearly > 1.6 indicating 4 copies of these sequences on the sSMC. **c. **HumanCytoSNP-12 result. Only chromosome 22 is depicted. The upper part shows the B-allele frequency (BAF) and the lower part shows the Log R Ratio along chromosome 22. Based on the BAF (presence of a BAF of 0.5 in at least a part of the maternally inhereted sSMC, indicating the presence of a meiotic tetrasomy), the MLPA result of a partial tetrasomy 22q11, is confirmed.

In **case 25 **of an extra familial del(22)(q11.2) the results of MLPA with the 22q11 probes in the four kits were indicative for 4 copies of this chromosomal region: relative probe signals were 1.770 and 1.621 (P181), 1.760 and 1.869 (P182), 1.688 (P036B) and 1.806 (P070) (Figure 2b). This contradicts the FISH results with one signal on the sSMC for two different 22cen-probes, one for the rDNA-probe and one signal with the probe from the Cat Eye Syndrome (CES)-region (Figure 2a). HumanCytoSNP-12-analysis confirmed the sSMC to be at least a partial duplication of chromosome 22, indicated by a BAF of 0.5, resulting in 4 copies of the sequences detected by the proximal 22q-probes in the 4 MLPA kits (Figure 2c).

### b. Mosaic cases

In one out of four mosaic cases (case 3), MLPA correctly identified four copies of 9ptel (P070, P036B), 9p11 (p182) and 9p13.2 (P181) with relative probe signals > 1.6 (table 3). However, in three out of four mosaic cases (cases 4, 6 and 20), the relative probe signals of some probes were below the expected values (1.3 for a trisomy and 1.6 for a tetrasomy). In these cases, the level of mosaicism, which was determined in cultured AF cells, was unknown in uncultured AF cells from which the DNA for MLPA was isolated. Nevertheless, the sSMC in all three cases could be identified by making use of our own calculated cut off values (table 2) with relative probe signals of the involving probes above the normal cut off values in all kits.

### Unique sequence negative sSMC

The unique sequence negative sSMC (cases 2, 8-10, 15-19, 26-30) all showed normal MLPA results with relative probe signals between the normal cut off values 0.7 and 1.3 confirming GTG/FISH results. There were no false positive cases.

## Discussion

sSMC, when detected with conventional cytogenetic banding techniques, are still a problem as they often are too small or without a specific banding pattern to be considered for their chromosomal origin by traditional banding techniques. Therefore, molecular cytogenetic techniques are often needed for further characterisation. Since the phenotypic consequences of a sSMC will greatly depend on its genetic content and chromosomal origin, it is of particular clinical importance to rapidly distinguish unique sequence-negative from unique sequence-positive sSMC because the former are less likely to be associated with an abnormal fetal outcome. For a collection of all available reported sSMC cases, see the sSMC database [22].

Different papers describe the use of genomic array analysis for identification of sSMC [15,16,23,24]. The big advantage of this technique is that the exact chromosomal content of the unique-sequence positive sSMC can be determined in one reaction, although targeted FISH is often used following an abnormal array result and is sometimes necessary to determine the structure of the sSMC [24]. However, genomic array analysis is labour intensive, time-consuming and expensive. The use of the MLPA technique for characterisation of some specific sSMC has already been described in postnatal cytogenetics [25-28]. In this paper, we show that MLPA with centromere and telomere kits may be a quick initial approach for sSMC characterisation in prenatal diagnosis when time is limited. From every AF sample that we receive in our laboratory 4 ml is used for direct DNA isolation. As soon as a sSMC is found, this DNA can be used for MLPA and results are available within less than 24 hours. In case of a positive result, targeted FISH and/or array on (un)cultured AF cells may be used for confirmation and further identification. However, as with array analysis, MLPA will give normal results in case of an unique sequence-negative sSMC or in case of low level mosaicism [16]. Therefore, other techniques such as cenM-FISH [8] or sequential targeted FISH [29], although labour-intensive and frequently time-consuming, will be necessary for determining the chromosomal origin. Since such a sSMC most probably does not contain euchromatin, in the meanwhile, the parents can be karyotyped after reassurance.

Despite the development of molecular techniques for sSMC identification, conventional staining techniques are still valuable. Since 35% of the marker chromosomes with a known chromosomal origin are derived from chromosome 15 [30], the first thing to do in case of a satellited sSMC is a DA-DAPI staining. If positive, targeted FISH with chromosome 15 specific probes can be applied for further identification. If negative, FISH with a 13/21 and 14/22 centromere probe in conjunction with subcentromere probes for these 4 chromosomes can quickly elucidate the chromosomal content of the sSMC. In case of a non-satellited sSMC, MLPA might be a rapid and rather non-expensive technique for distinguishing between an unique sequence-positive and -negative sSMC.

The centromere kits P181 and P182 are presented by MRC Holland as kits for identification of sSMC. However, we recommend using the telomere kits in addition to these centromere kits for sSMC identification for three reasons. Firstly, by using only the centromere kits, the neo(12) and neo(15) would both have been missed. This type of sSMC, first described by Blennow [31] is rare. Up till now about ~90 neocentric acentric marker chromosomes have been described in patients with idiopathic mental retardation but also in cancer cells [32,33]. Secondly, the sSMC in cases 7 and 11 could be correctly identified as being unbalanced translocations by using centromere concomitant with telomere kits. Although rare, this type of sSMC, the so-called unique complex sSMC which are derived from more than one chromosome, may be underdiagnosed as suggested by Trifonov et al. [34], and MLPA with centromere and telomere kits may enhance their detection rate. And finally, since most sSMC are derived from the acrocentric chromosomes, the simultaneous use of centromere and telomere kits allows for more markers to be tested in the subcentromeric region since the telomere kits also contain probes in the proximal long arm of the acrocentric chromosomes instead of a specific short arm subtelomere probe which they lack.

In 5/6 mosaic cases with the level of mosaicism determined in cultured cells, DNA isolated from uncultured AF cells was used for MLPA hampering the interpretation of the results. For instance, normal MLPA results in cases 18 and 19 are most probably explained by absence of unique sequences on both sSMC. However, due to low-level mosaicism at least in the AF cell cultures and therefore probably also in the uncultured AF cells used for MLPA, a normal result caused by low-level mosaicism can never be excluded. It is known that discrepancies may exist concerning mosaicism level between cultured and uncultured AF cells with the level often being higher in uncultured cells since these are not subjected to selection, mostly in favour of normal cells as seen in cell cultures. However, the reverse has also been observed in some cases of tissue specific mosaicism if the contribution of the affected organ or organsystem to the total AF cell population is small [35], but also in cases of generalised mosaicism [36]. From this experience, we learned that it is important to make FISH-slides at the time of DNA isolation necessary for determination of the level of mosaicism in the DNA sample enabling a proper interpretation of molecular results.

In cases 13 and 25 we show that the use of molecular techniques such as MLPA and array analysis may show that some sSMC are much more complex than initially thought on the basis of conventional banding techniques and FISH. At the moment we are performing further FISH studies in order to elucidate the exact structure of the sSMC in both cases. Tsuchya et al. [24] already published the uncoverage of unexpected complexity in the form of complex rearrangements of some sSMC when they used array CGH. This will ultimately lead to a more accurate sSMC-phenotype correlation which is important for proper counselling of the prospective parents when the sSMC is found prenatally.

## Conclusion

In this paper we show that MLPA with centromere (P181 and P182)-and telomere (P036B and P070)-kits allow for the rapid differentiation between unique sequence positive and negative sSMC. As compared to multicolour FISH techniques and microarray analysis, MLPA is a rather non-expensive and easy to perform technique in most clinical cytogenetic laboratories for the rapid elucidation of the harmfulness of a prenatally detected sSMC with results available within 24 hours.

## Competing interests

The authors declare that they have no competing interests.

## Authors' contributions

DVO coordinated the study and wrote the paper. MB performed the MLPA and microarray analyses. PN performed cytogenetic and FISH analyses. MIS, DVO, GH and R-JHG were responsible for the final (molecular) cytogenetic diagnoses and reports. GH and DVO studied the literature. R-JHG coördinated the genetic counselling of the parents. All authors read and approved the manuscript.

## Supplementary Material

Additional file 1 **Supplemental Table**. 29 prenatal cases with a sSMC: indication, sSMC identification with conventional staining and FISH techniques, positive or negative for euchromatin and final karyotype. Click here for file
