# Supplementary material for: Multiplex ligation dependent probe amplification (MLPA) for rapid distinction between unique sequence positive and negative marker chromosomes in prenatal diagnosis
Source: Mol Cytogenet. 2011 Jan 14;4:2. doi: 10.1186/1755-8166-4-2 (PMC3033356; doi:10.1186/1755-8166-4-2)
Supplement: Additional file 1 — Supplemental Table. 29 prenatal cases with a sSMC: indication, sSMC identification with conventional staining and FISH techniques, positive or negative for euchromatin and final karyotype. [file 1755-8166-4-2-S1.DOC]

Additional file 1: 29 prenatal cases with a sSMC: indication for prenatal diagnosis, sSMC identification (with conventional staining and FISH techniques), positive or negative for euchromatin and final karyotype.

| Case | Indication | sSMC identification | Euchromatin | Karyotype |
| --- | --- | --- | --- | --- |
|  |  |  |  |  |
| 1^1^ | US abnormalities (intrauterine growth retardation) and AMA36 | **FISH:**  -wcp3 +  -pα3.5 (3cen) +  -RP11-662C7 (3p11.1) +  -RP11458D16 (3p11.1) +  -RP11-91A15 (3p11.1) +  -RP5-1186B18 (3ptel) –  -RP1-196F4 (3qtel) –  -CTD2005N19 (3q11.2) –  -CTD-2007O24 (3q11.2) –  -RP11-21I16 (3q11.2) –  **Polymorphic DNA marker studies:**  Breakpoint p-arm between D3S3681 and D3S3633 at 3p12.2. | pos | -In STC-villi: 47,XX,+3  -In LTC-villi and AF cells: 47,XX,+der(3)(:p12.2🡪cen:) |
| 2 | AMA38 | **DA/DAPI:**  Negative  **FISH:**  Sequential FISH with almost all cen-probes with pYAM11-39 (**4cen**) being **positive**.  (pX2(2cen)-, pα3.5(3cen)-, pG-A16(19/5/1cen)-, D6Z1(6cen)-, pα7t1(7cen)-, αp8(8cen)-, pH8(10cen)-, pα12H8(12cen)-, L1.26(13/21cen)-, c237(14/22cen)-, p17H8(17cen)-, L1.84(18cen)-, CEP20(20cen)-XB12(Xcen)-, pDP97(Ycen)-)  -wcp4 –  -RP11-109P3 (4p11p12) –  -RP11-702A23) (4p11) –  -RP11-1115N1 (4q12) –  -RP11-61F5 (4q12) –  -RP1-36P21 (4pter) – | neg | 47,XX,+min(4)(:p11🡪q11:) |
| 3 | AMA + abnormal first trimester screening (risk 1:10) | **FISH:**  -wcp9 +  -pHuR98 (9het) +  -CEP9 (9 cen) x2  -RP11-151G22 (9q13) –  -RP1-43N6 (9ptel) x2  -CTB-135I17 (9qtel) – | pos | STC-villi: 46,XX[8]  LTC-villi: 47,XX,+psu idic(9)(q12)[14]/46,XX[2] |
| 4 | US abnormalities (hernia diaphragmatica left) | **FISH**;  -wcp12 **+**  -pα12H8 (12cen) **+**  -CTB-124-K20 (12ptel) x2  -RP1-221K18 (12qtel) – | pos | 47,XX,+i(12)(p10)[17]/46,XX[2] |
| 5 | US abnormalities (hernia diaphragmatica, ventriculomegalie, Arnold Chiari malformation, echodense kidneys) | **FISH:**  -wcp12 **+**  -pα12H8 (12cen) **+**  -CTB-124K20 (12ptel) x2 | pos | 47,XX,+i(12)(p10) |
| 6 | US abnormalities (left hydroureter, left hydronefrosis) | **FISH**:  -pα12H8 (12cen) **–**  -RP11-73I12 (12p11.21) –  -CTB-124K20 (12ptel) x2  -LSI TEL AML1 (12p13.2) x2 | pos | 47,XY,+neo(12)(pter->p12.3:)[16]/46,XY[18] |
| 7 | US abnormalities (polyhydramnion, unilateral hydrothorax) | **Parental karyotyping:** t(4;13)(q31.3;q13)mat | pos | 47,XX,+der(13)t(4;13)(q31.3;q13)mat |

| 8 | US abnormalities (hygroma colli, single umbilical artery) | **FISH**:  -L1.26 (13/21cen) +  -RP11-1144F2 (21q11.2) –  -CTD-2349B11 (13q12) –  -wcp13 –  -wcp21 –  -r521 (r521) – | neg | 48,XXY,+min(13 or 21) |
| --- | --- | --- | --- | --- |
| 9 | Abnormal first trimester screening (risk 1:219) and paternal marker chromosome | **FISH:**  -r521 (rDNA) x2  -L1.26 (13/21cen) +  -RP11-1008N13 (21q11.2) –  -CTD2349B11 (13q12.1) –  -D15Z1 (15psatIII) – | neg | 47,XX,+inv dup(13 or 21)pat |
| 10 | Abnormal first trimester screening (risk 1:25) | **FISH**:  -c237 (14/22cen) +  -r521 (rDNA) x2  -p190.22 (22cen) –  -RP11-516D7 (14q11.2) –  -RP11-958H20 (22q11) –  -L1.26 (13/21cen) –  -pTRA-20 (15cen) –  -D15Z1 (15psatIII) +^2^ | neg | 47,XY,+inv dup(14)(q11.2)mat |
| 11 | US abnormalities (pes equinovarus bilateral, overlapping fingers, intrauterine growth retardation) | **Satellited sSMC: DA-DAPI:** negative  **FISH**:  -r521 +  -wcp14 +  -wcp22 –  -102D10 (22q11, CES) –  -WCP16 + | pos | 47,XX,+der(14)t(14;16)(q12;q21) |
| 12 | US abnormalities (intrauterine growth retardation) | **FISH:**  -r521(rDNA) x2  -pTRA-20 (15cen) x2  -SNRPN (15q11.2) x2 | pos | 47,XX,+inv dup(15)(q12) |
| 13 | US abnormalities (Dandy Walker malformation) | **C-, DA/DAPI- and NOR-banding:** negative  **FISH:**  -wcp15 +  -L1.26 (13/21cen) –  -p22/1:2.1 (22cen) –  -pTRA-20 (15cen) –  -CRN189-1 (15q11.2) –  -p80 (15q25-qter) x2 | pos | 47,XX,+neo(15)(qtel->q2?4:)^3^ |
| 14 | US abnormalities (ascites), AMA | **Parental karyotyping:**  Mother carrier t(9;15)(p12;q14)  **FISH:**  -wcp15 +  -pTRA-20 (15cen) +  -SNRPN +  -P11-622N4 (9p13.1) +  -RP1-43N6 (9ptel) +  -CEP9 (9cen) –  -RP11-360J18 (15q13.1) + | pos | 47,XX,+der(15)t(9;15)(p12;q14)mat |
| 15 | AMA 36 | **DA/DAPI:** positive  **FISH:**  -pTRA-20 (D15Z4,15cen) x2  -pTRA-25 (D15Z3, 15cen) x2  -D15Z1 (15psatIII) x2  -r521 (rDNA) x2  -Y41 (15q11.2, D15S11) **–**  -Y11H11 (15q11.2) –  -SNRPN (15q11.2) – | neg | 47,XY,+inv dup(15)(q11) |

| 16 | Risk NTD | **FISH:**  -pTRA-20 (D15Z4,15cen) x2  -pTRA-25 (D15Z3, 15cen) x2  -D15Z1 (15psatIII) x2  -r521 (rDNA) x2  -Y41 (prox SNRPN) **–**  -wcp15 –  -L1.26 (13/21cen) **–**  -c237(14/22cen) **–** | neg | 47,XY,+inv dup(15)(q11.2)pat |
| --- | --- | --- | --- | --- |
| 17 | Recurrence risk of tris 21 | **DA/DAPI:** positive  **FISH:**  -D15Z1 (15psatIII) x2  -pTRA-20 (D15Z4, 15cen) x2  -r521 (rDNA) x2  -SNRPN (15q11.2) – | neg | 47,XY,+inv dup(15)(q11.2)mat |
| 18 | AMA | **DA-DAPI:** positive  **FISH:**  -D15Z1(15psatIII) –  -r521 (rDNA) –  -SNRPN (15q11.2) –  -pUC1.77 (1qhet) –  -pHuR98 (9qhet) –  -pHuR195 (16qhet) **+**  -RP11-388M20 (16p11.2) –  -RP11-474B12 (16q12.2) – | neg | mos 47,XX,+min(16)(:p11.1->q11.1:)[6]/46,XX[14] |
| 19 | AMA 39 | **-SKY**: #4, 11, 3, 17  **-FISH**:  -p17H8 (17cen) **+**  -wcp17 **–**  -RP11-1149K20 (17p11.2) **–**  -RP11-252O24 (17q11.2) **–** | neg | mos 47,XX,+min(17)(:p11.1->q11.1:)[10]/  46,XX[12] |

| 20 | Half of twin with the other foetus being carrier of a mar(20)(investigated elsewhere) and AMA 39 | **FISH:**  **-**wcp20 +  -CEP20 (20cen) + | pos | mos 47,XY,+r(20)(q11.21q13.12)[20]/46,XY[3] |
| --- | --- | --- | --- | --- |
| 21 | AMA 36 | **FISH:**  -c237 (14/22cen) x2  -p190.22 (22cen) x2  -M51 (DGSCR, 22q11) **+**  -102D10 (CES, 22q11) **+**  -L1.26 (13/21cen) **–**  -pTRA-20(D15Z4,15cen) **–** | pos | 47,XX,+inv dup(22)(q11.21) |
| 22 | US abnormalities (hypoplastic right heart syndrome, single umbilical artery, echodense intestines). | DA/DAPI – **FISH:**  -r521(rDNA) x2  -p190.22 (22cen) x2  -102D10 (CES,22q11) x2  -M51(DGSCR) – | pos | 47,XY,+inv dup(22)(q11.21) |
| 23 | AMA 36 | DA/DAPI – **FISH:**  -r521(rDNA) x2  -c237 (14/22cen) x2  -RP11-958H20 (22q11.1) **+**  -102D10 (CES,22q11) **+**  -M51 (DGSCR, 22q11) **–**  -RP11-90D17 (22q12.2) **–**  -L1.26 (13/21cen) **–**  -RP11-26P6 (13q12.11) **–**  -RP11-1144F2 (21q11) **–**  -CTD-2349B11 (13q12.11) **–** | pos | 47,XX,+inv dup(22)(q11.21) |
| 24 | US abnormalities (intra-uterine growth retardation) and recurrence risk inv dup (22)(q11.2)mat | **FISH**  -p190.22 (22cen) x2  -102D10 (22q11.2)(CES) x2 | pos | 47,XX,+inv dup(22)(q11.2)mat |

| 25 | Recurrence risk of maternal marker chromosome (del(22)(q11.2) in 9% of lymphocytes) | **FISH**  -p190.22 (22cen) +  -c237 (14/22cen) +  -r521 (rDNA) +  -102D10 (22q11.2)(CES) +  -M51 (DGSCR) –  -122B5 (DGSCR) –  -SC11.1 (DGSCR) – | pos | 47,XY,+del(22)(q11.2)mat |
| --- | --- | --- | --- | --- |
| 26 | AMA 36 | DA/DAPI – **FISH:**  -r521(rDNA) x2  **-**p190.22 (22cen) x2  -M51(DGSCR, 22q11) –  -D22S181 – | neg | 47,XX,+inv dup(22)(q11.1)pat |
| 27 | US abnormalities (bilateral cleft lip) | **FISH**:  -r521 (rDNA) x2  -p190.22 (22cen) x2  **-**102D10 (CES, 22q11.2) **–**  -c237 (14/22cen) **–**  -RP11-516D7 (14q11.2) **–**  -RP11-777M14(14q11.2) –  -L1.26 (13/21cen) **–**  -pTRA-20 (D15Z4,15cen) **–** | neg | 47,XY,+inv dup(22)(q11.1)pat |
| 28 | AMA38 | **DA/DAPI –**  **FISH:**  -r521 (rDNA) x2  -p190.22 (22cen) x2  -102D10 (CES, 22q11.2) **–** | neg | 47,XY,+inv dup(22)(q11)mat |

| 29 | AMA | **DA/DAPI –**  **FISH:**  -r521 (rDNA) x2  -p190.22 (22cen) +  -102D10 (CES, 22q11.2) **–**  -RP11-155N18 (22q11.1) –  -RP11-958H20 (22q11.1) –  -RP11-318B11 (22q11.1) –  -RP11-652F11 (22q11.21) – | neg | 47,XX,+inv dup(22)(q11) |
| --- | --- | --- | --- | --- |

AMA= advanced maternal age; US= ultrasound abnormalities; STC-villi= short-term cultured villi; LTC-villi= long-term cultured villi; AF= amniotic fluid

^1^ This case was previously published by Srebniak et al. [39].

^2^D15Z1 was positive on both normal chromosomes 15 at 15p and on the sSMC that showed to be derived from chromosome 14. It is known that D15Z1 often hybridises to the short arm of other acrocentric chromosomes, especially chromosome 14 [38].

^3^ This case was published earlier by Van Opstal et al. [40].
